# Physicochemical Properties, Antioxidant and Antidiabetic Activities of Polysaccharides from Quinoa (*Chenopodium quinoa* Willd.) Seeds

**DOI:** 10.3390/molecules25173840

**Published:** 2020-08-24

**Authors:** Minghui Tan, Senlin Chang, Jianing Liu, Hang Li, Pengwei Xu, Peidong Wang, Xiaodong Wang, Mingxia Zhao, Bing Zhao, Liwei Wang, Qingsheng Zhao

**Affiliations:** 1 Division of Bioresources and Health Product Engineering, State Key Laboratory of Biochemical Engineering, Institute of Process Engineering, Chinese Academy of Sciences, Beijing 100190, China; mhtan@ipe.ac.cn (M.T.); changsenlin14@mails.ucas.edu.cn (S.C.); liujianing17@mails.ucas.edu.cn (J.L.); lihang18@mails.ucas.edu.cn (H.L.); xupengwei17@mails.ucas.edu.cn (P.X.); wangpeidong19@mails.ucas.ac.cn (P.W.); xdwang@ipe.ac.cn (X.W.); mxzhao@ipe.ac.cn (M.Z.); bzhao@ipe.ac.cn (B.Z.); 2 School of Chemical Engineering, University of Chinese Academy of Sciences, Beijing 100049, China

**Keywords:** *Chenopodium quinoa* Willd, polysaccharide, fractional precipitation, antioxidant, antidiabetic

## Abstract

Quinoa is known for its rich nutrients and bioactive compounds. In order to elucidate the preliminary structural characteristics and biological activity of polysaccharides from quinoa (QPs), five crude polysaccharides (QPE50, QPE60, QPE70, QPE80 and QPE90) were successively fractionated by gradient ethanol, and their physicochemical properties, antioxidant and antidiabetic activities were analyzed. The results implied that their total sugar contents were 52.82%, 63.69%, 67.15%, 44.56%, and 41.01%, and their weight-average molecular weights were 13,785 Da, 6489 Da, 4732 Da, 3318 Da, and 1960 Da, respectively. Glucose was a predominantly monosaccharide in these QPs, which together in QPE50, QPE60, QPE70, QPE80, and QPE90, respectively, made up 94.37%, 87.92%, 92.21%, 100%, and 100% of the total polysaccharide. Congo red test showed that all five QPs contained triple-helix structure. The Fourier transform-infrared spectroscopy (FT-IR) and X-ray diffractometry (XRD) results suggest that the QPs form a semi-crystalline polymer constituted typical functional groups of polysaccharide including CO, CH and OH. The thermogravimetric analysis (TGA) of QPs showed that weight loss was at about 200 °C and 320 °C. The observation from scanning electron microscope (SEM) and atomic force microscope (AFM) image indicated that the morphology of QPs exhibited spherical shape. Antioxidant and antidiabetic assay exhibited that all five QPs samples had certain antioxidant and antidiabetic activities, and QPE90 showed the best antioxidant and antidiabetic activity. Overall, QPs present a promising natural source of food antioxidants and antidiabetic agents.

## 1. Introduction

Quinoa, is a crop native to the Andean region. It is an annual dicotyledonous herb belonging to the genus *Chenopodium*, which has been cultivated for 5000–7000 years [1]. The seeds are in the shape of oval pills, and the colors are purple, red, yellow or green. The seed coat is covered with a layer of water-soluble saponins [2], so it has a slightly bitter taste. Quinoa originated in the Andean region of South America and currently grown in Bolivia, Peru, United States, Ecuador and Canada as a food crop [3]. Quinoa is rich in protein, carotenoids and Vitamin C. Its protein has a balanced composition of amino acids, with a high content of lysine (5.1–6.4%) and methionine (0.4–1.0%) [4]. The seed is also rich in mineral nutrients, such as Ca, Fe, Zn, Cu and Mn. Among them, the content of Ca (874 mg/kg) and Fe (81 mg/kg) is significantly higher than that of most common cereals [5]. Therefore, quinoa is called “nutritional gold”, “super grain” and “future food” by international nutritionists [6]. Quinoa has starch polysaccharides and non-starch polysaccharides. Starch is the predominant carbohydrate in quinoa seeds with a content of 58.1–64.2%. It is composed of amylose and amylopectin. The amylopectin content is higher, accounting for 93–94% of the total starch content [7]. Non-starch polysaccharide contains cellulose, hemicellulose and pectin mainly. As a class of non-starch polysaccharides, dietary fiber is rich in quinoa, accounting for 7–9.7%, of which insoluble dietary fiber accounts for 78%. Insoluble fiber from quinoa is mainly composed of galacturonic acid, galactose, xylose and glucose, while soluble fiber is mainly composed of glucose, galacturonic acid and arabinose [8].

Polysaccharide is a kind of polymer composed of more than 10 monosaccharides connected together by different types of glycosidic bonds. It is one of the most indispensable biological macromolecular substances in life’s activities. In the past two decades, a large number of studies have found that non-starch natural polysaccharides isolated from various biological sources, such as animals, plants, microorganisms and algae have rich biological activities, such as immune regulation, anti-tumor, anti-oxidation, anti-radiation, anti-virus, hypolipidemic, hypoglycemic and antibacterial activities [9]. The molecular structure and spatial conformation of polysaccharides usually carry a lot of biological information and play a vital role in the life activities of organisms [10]. Among them, the triple helix polysaccharide is particularly noticeable. It was reported that polysaccharides, with triple-helical conformation, usually have good biological activity, such as lentinan [11], kelp polysaccharide [12], *Schizophyllum* polysaccharide [13], and *Dianthus longifolia* polysaccharide [14], etc. With the improvement in polysaccharide separation, purification and structural analysis technology, studies focuses on the structure and function of natural active polysaccharides have been continuously deepened, and polysaccharides have been found to have great research and utilization value in clinical medicine and health care product development. That is why polysaccharides become hot topics in the fields of natural medicine development and health food development.

Current reports on quinoa mainly focus on the study of protein, saponins and phenolic acids. However, there are few reports on the physicochemical and biological activity of quinoa polysaccharides. Therefore, the aim of this work was to evaluate the relationship between structural characteristics and biological activities of quinoa polysaccharide, which might provide useful guidance for exploiting quinoa polysaccharide for utilization in processed foods. 

## 2. Results and Discussion

### 2.1. Chemical Compositions of QPs

The yield and chemical component of QPs prepared by ethanol fractional precipitation are shown in Table 1. The yield of QPs was in the following order from low to high: QPE90 < QPE50 < QPE80 < QPE60 < QPE70. Using glucose as the standard, the total sugar content of QPE70 was estimated to be 67.15%, which was higher than that of QPE90 (41.01%), QPE80 (44.56%), QPE50 (52.82%) and QPE60 (63.69%).

The content of uronic acid in polysaccharide could enable polysaccharide to be negatively charged, which had an important effect on the biological activity of polysaccharide [15]. From the above result, QPE70 had the highest uronic acid content compared with other QPs sample.

Molecular weight is an important feature of polysaccharides, which is closely related to the structure and biological activity of polysaccharide. The average molecular weight of QPs obtained by gradient ethanol precipitation gradually decreased as the ethanol concentration increased (Table 1). In addition, the polydispersity index (Mw/Mn) of five polysaccharides were less than 2, which means that molecules were less dispersed in the aqueous solution [16]. Furthermore, the polysaccharide molecules start to dehydrate when ethanol was added to a solution containing polysaccharide, and then polysaccharide conformation was transformed and assembled, due to the enhancement of intramolecular hydrogen bonding [17]. As reported in the literature, different average molecular weights of polysaccharide fractions could be precipitated by gradient ethanol [18].

### 2.2. Monosaccharide Composition of QPs

**Figure 1:**
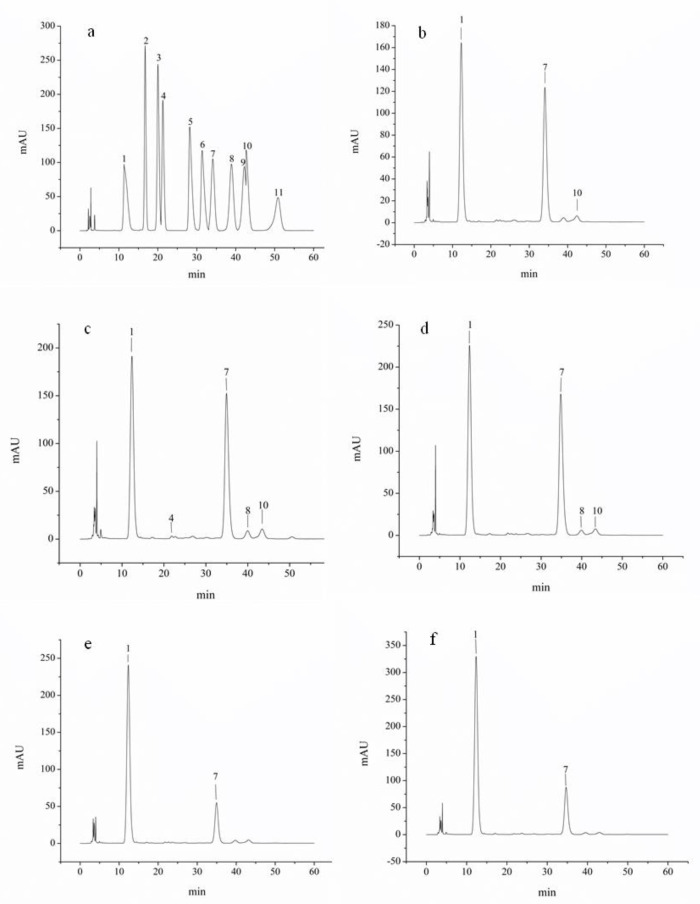
HPLC chromatogram of standard monosaccharide and QPs (**a**) standard monosaccharide, (**b**) QPE50, (**c**) QPE60, (**d**) QPE70, (**e**) QPE80, (**f**) QPE90 (1. PMP, 2. Mannose, 3. Ribose, 4. Rhamnose, 5. Glucuronic acid, 6. Galacturonic acid, 7. Glucose, 8. Galactose, 9. Xylose, 10. Arabinose, 11. Fucose).

The biological activity of polysaccharide is usually affected by their monosaccharide compositions. The monosaccharide compositions of different QPs were analyzed by HPLC (Table 2 and Figure 1). According to the monosaccharide standards, QPE50 was composed of glucose and arabinose with the molar ratio of 94.37:5.63, QPE60 was composed of rhamnose, glucose, galactose and arabinose with the molar ratio of 0.73:87.92:4.67:6.68, QPE70 was composed of glucose, galactose and arabinose with the molar ratio of 92.21:2.98:4.81, while QPE80 and QPE90 were all composed of glucose. Glucose was a predominantly monosaccharide in these QPs, which was consistent with the results from previous report [19].

### 2.3. Congo Red Test

The polysaccharide sample with a triple-helix structure, can form a complex with Congo red under weak alkaline conditions, and its UV absorption will be red-shifted compared to Congo red. With the NaOH concentration gradient increased in the reaction system, the hydrogen bond between polysaccharide molecules broken, and the triple-helix structure decomposed into a single strand, which could not form a complex with Congo red and became a random coil, and λmax decreased sharply. It was reported that polysaccharide with triple helixes have high biological activity [20].

**Figure 2:**
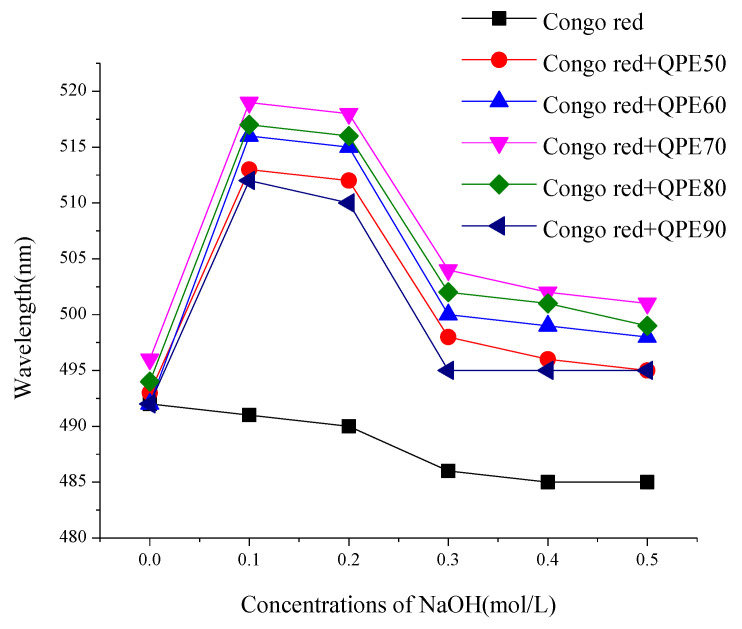
Congo red test results of QPs.

The results of the Congo red assay for each sample of QPs are shown in Figure 2. It can be seen from the figure that under weak alkaline conditions (NaOH concentration less than 0.1 mol/L), the λmax of QPs mixed with Congo red showed a significant redshift. However, the λmax decreased sharply as the continuously increase of NaOH concentration. For the blank control group without polysaccharide, λmax also decreased as the continuously increase of NaOH concentration. These results suggested that there was a triple-helix structure in all QPs samples.

### 2.4. UV and FT-IR Spectroscopy of QPs

**Figure 3:**
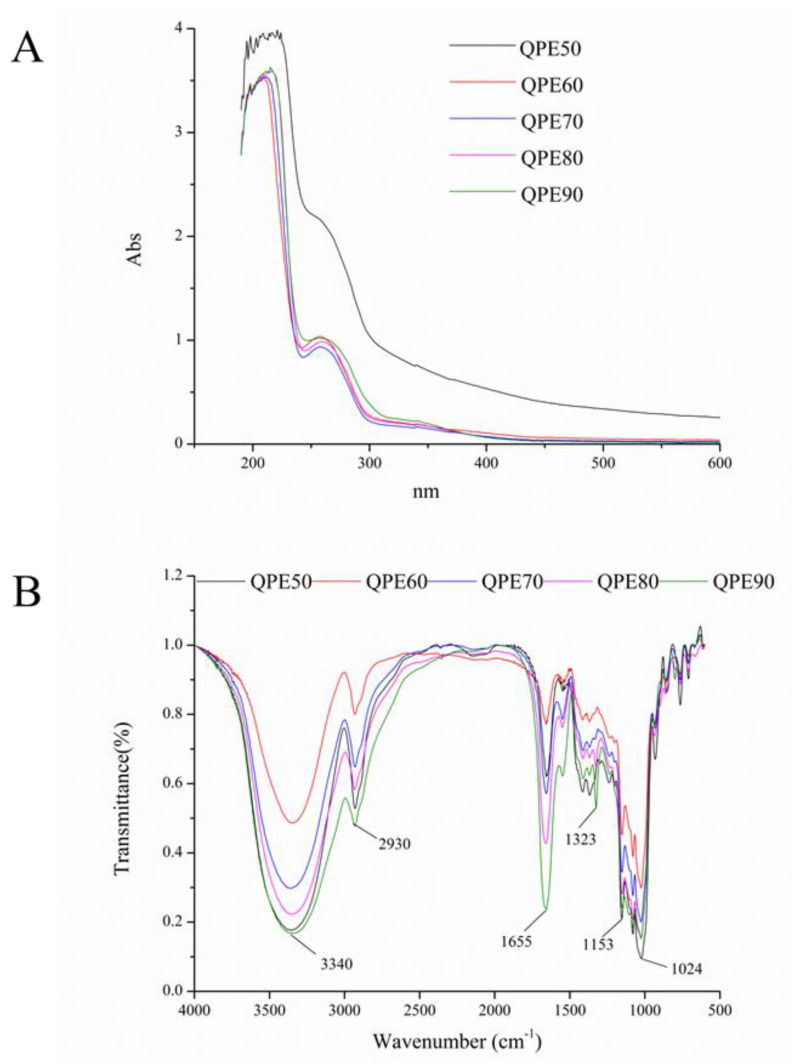
Ultraviolet spectrum (**A**) and FT-IR spectrum (**B**) of QPs.

The ultraviolet spectrum of QPs obtained by gradient ethanol precipitation is shown in Figure 3A. It could be observed that there was an obvious absorption peak around 280 nm in all QPs samples, indicating that protein was the component, which was consistent with the polysaccharide component analysis.

The FT-IR spectrum of QPs is shown in Figure 3B, in which the typical absorption of polysaccharide could be observed. It could be found that QPE50, QPE60, QPE70, QPE80, QPE90 had similar absorption band from 4000 cm^−1^ to 400 cm^−1^. The absorption peak around 3340 cm^−1^ was contributed by –OH stretching vibration, demonstrating the existing of –OH. The weak absorption around 2930 cm^−1^ was C-H stretching vibration, including –CH_2_, –CH_3_. The strong peak at approximately 1653 cm^−1^ belonged to the –C=O stretching vibration, while the peak at around 1153 cm^−1^ indicated the existence of pyranose, which was matched the result of monosaccharide composition analysis. Furthermore, the peak around 920 cm^−1^ was the representative absorption of β configuration [21]. The FT-IR spectra of QPs were characteristic absorption peaks of polysaccharides, which may be consistent with β-glucans.

### 2.5. SEM Analysis

**Figure 4:**
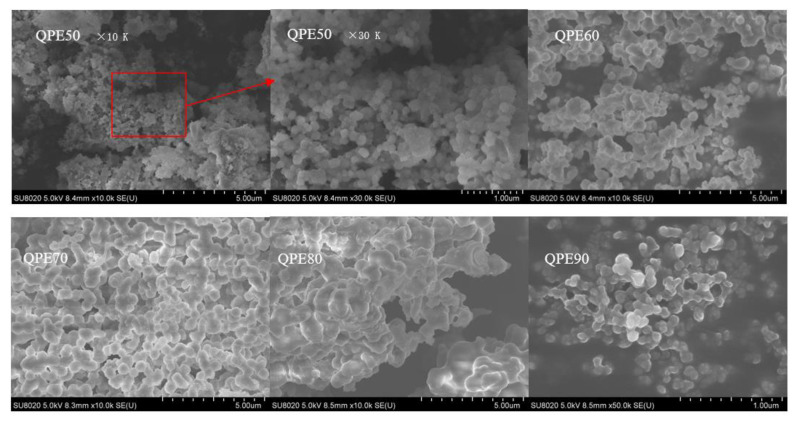
SEM images of QPs.

The SEM images of QPs are shown in Figure 4, which showed the samples morphology. From the above molecular weight analysis, it could be found that the molecular weight of QPE-50, QPE-60, QPE-70, QPE-80, QPE-90 was gradually decreasing. We can see that QPs particles shrink with the reduction of molecular weight. The particles were separated from each other. This phenomenon could be explained by hydrogen bonds exist in polysaccharide. Polysaccharide with higher molecule weight are more likely to form hydrogen bonds between polysaccharides, while polysaccharides with low molecule weight tend to form hydrogen bonds with water. Polysaccharides are dissolved in water through the formation of hydrogen bonds between polysaccharide and water. When ethanol was added to a solution containing polysaccharide, polysaccharide with high molecule weight were rapidly precipitated, due to the formation of a large number of intermolecular hydrogen bonds. Therefore, they exhibit a relatively aggregated state, while polysaccharide with low molecule weight are more likely to form hydrogen bonds, with water and difficult to aggregate with each other, so as to show a relatively dispersed state.

### 2.6. AFM Analysis

**Figure 5:**
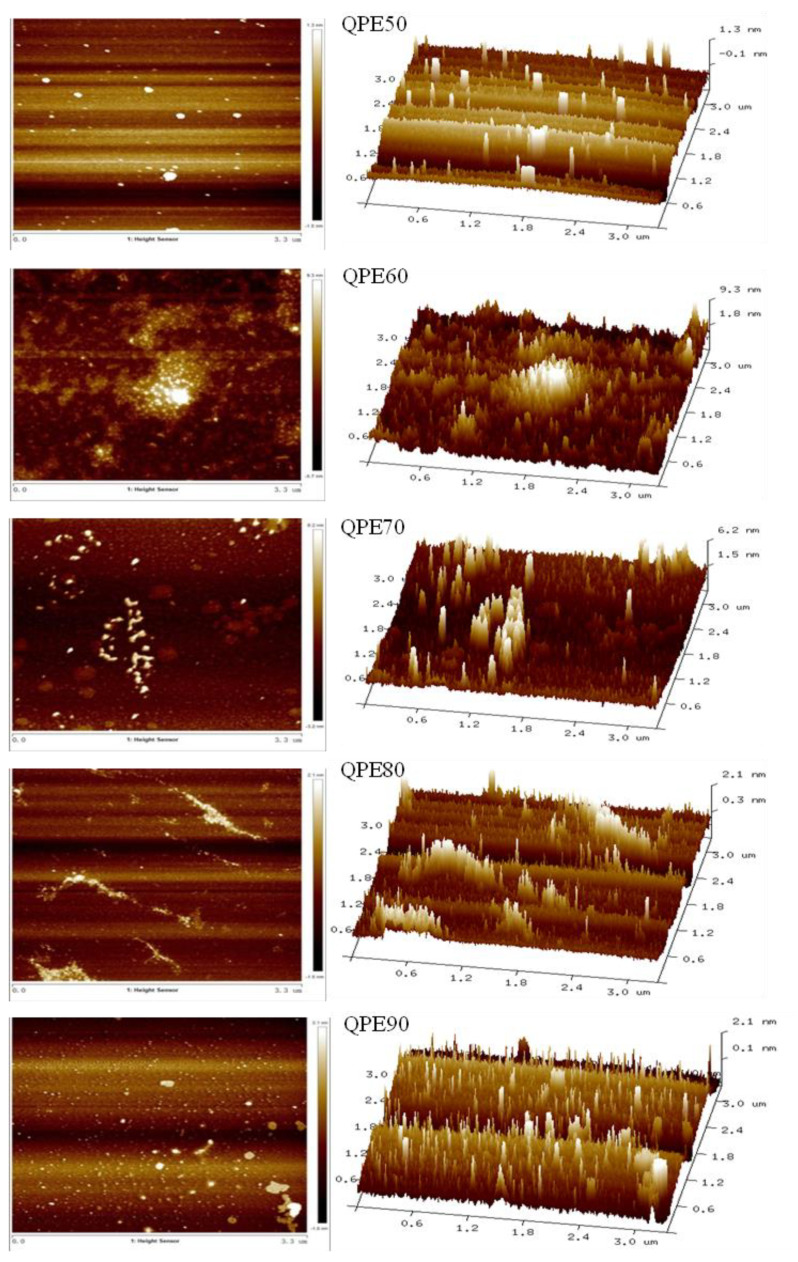
Atomic force microscope images (2D/3D) of QPs.

The AFM is a powerful technology to observe the molecular morphology of polysaccharides [22,23,24]. AFM images of QPs are shown in Figure 5. As can be seen from the images that apart from QPE80, all other QPs samples showed granular or spherical particles with irregular shape and size. The width of four polysaccharide chains were in the range of 0.146–0.166 μm, 0.101–0.123 μm, 0.072–0.096 μm and 0.052–0.079 μm, respectively. QPE80 appeared linear in structure and branched or coiled in water solution, the width of the polysaccharide chain was range from 0.022 to 0.046 μm, and the length was in the range of 0.737–1.728 μm. A helical conformation could be seen in the AFM image of QPE80.

Generally, the width of a single polysaccharide chain is between 0.1 and 1 nm [25]. From our results, the width of the QPs chain was more than 20 nm, which was much larger than a single polysaccharide chain, indicating that the polysaccharide unit could be branched and tangled with each other. As observed in the morphology of polysaccharide chains, QPs with higher Mw values had a wider polysaccharide chain, which was also due to their aggregation.

### 2.7. XRD Analysis

XRD technology is an important analytical tool to investigate the crystal structure of high molecular polymers. Generally, polysaccharides are relatively difficult to crystallize, and single crystal morphology is difficult to obtain using XRD analysis. According to reports in the literature, only some polysaccharides with rigid helix chains exist in crystalline form, due to their ordered structure [26].

**Figure 6:**
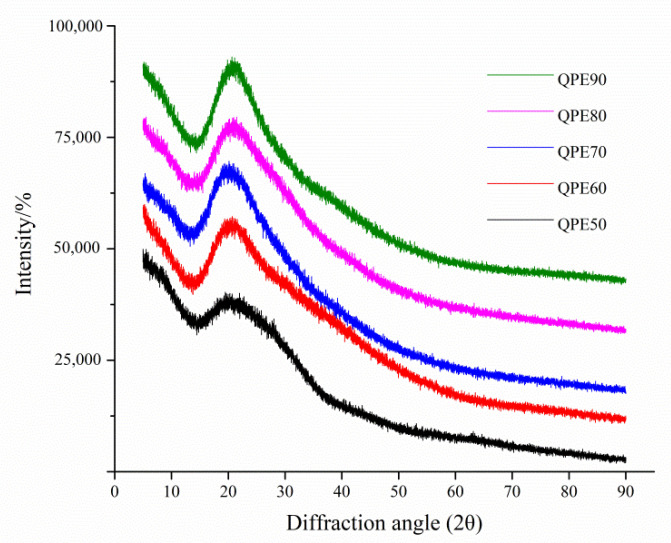
XRD patterns of QPs.

The XRD patterns recorded for QPs were between 5° and 90° (Figure 6). As can be seen from the figure, all five QPs showed low overall crystallinity and had a clear diffraction peak when 2θ was about 20.2°. This result revealed that the QPs were semi-crystalline polymers and possessed an ordered helical structure that could be related to their biological activity. According to the literature reported before [27,28], diffraction peaks were also observed in other polysaccharides when 2θ was about 20°. The crystal structure characteristics of polysaccharide directly determine its physical properties such as solubility, tensile strength, swelling, flexibility, or opaqueness [29]. In fact, the biological activity of polysaccharide is also affected by its crystal structure characteristics [30].

### 2.8. TG-DTA-DTG Analysis

Thermogravimetric analysis uses temperature as the abscissa and the weight loss rate of the sample as the ordinate, and records the weight change of the sample during the heating process to obtain the thermogravimetric curve, also known as the TG curve. Information, such as the thermal stability, thermal decomposition temperature, thermally stable temperature range, and composition of the substance can be obtained from the thermogravimetric curve. The DTG curve is obtained by taking the first derivative of the thermogravimetric curve against the temperature, and reflects the relationship between the rate of change of the sample mass and the temperature. The DTA method is a thermal analysis method to measure the temperature difference between sample and reference substance under the temperature condition of the program control.

**Figure 7:**
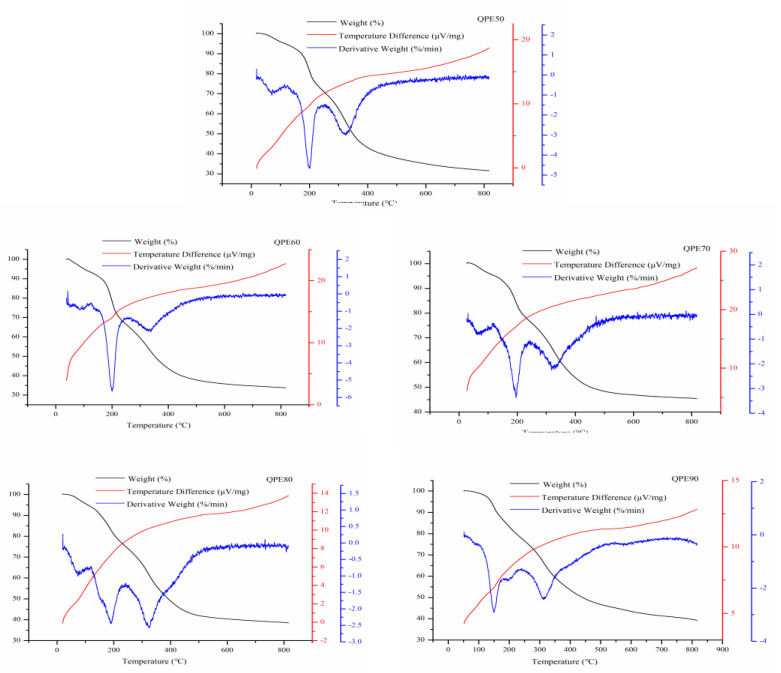
TG-DTA-DTG curves of QPs.

The TG-DTA-DTG results of QPs are shown in Figure 7. For QPE50, from the TG curve, there is a 7.41% weight loss in the range of 50–150 °C, 54.73% weight loss in the range of 150–500 °C, and 6.09% weight loss in the range of 500–800 °C. DTG curve shows that there are two major weight loss stages, 150–250 °C and 250–400 °C. The maximum weight loss rate are at 200.98 °C and 325.11 °C, respectively. For QPE60, from the TG curve, there is a 8.7% weight loss in the range of 50–150 °C, 53.52% weight loss in the range of 150–500 °C, and 3.85% weight loss in the range of 500–800 °C. DTG curve shows that the maximum weight loss rate are at 200.28 °C and 337.41 °C in the two major weight loss stages. For QPE70, from the TG curve, there is a 6.98% weight loss in the range of 50–150 °C, 44.65% weight loss in the range of 150–500 °C, and 2.81% weight loss in the range of 500–800 °C. DTG curve shows that the maximum weight loss rate are at 195.98 °C and 318.18 °C in the two major weight loss stages. For QPE80, from the TG curve, there is a 9.4% weight loss in the range of 50–150 °C, 48.59% weight loss in the range of 150–500 °C, and 3.32% weight loss in the range of 500–800 °C. DTG curve shows that the maximum weight loss rate are at 188.86 °C and 324.16 °C in the two major weight loss stages. For QPE90, from the TG curve, there is a 7.6% weight loss in the range of 50–150 °C, 45.69% weight loss in the range of 150–500 °C, and 6.98% weight loss in the range of 500–800 °C. DTG curve shows that the maximum weight loss rate are at 149.37 °C and 313.27 °C in the two major weight loss stages. The DTA curve of all five QPs samples indicated that the decomposition of graded alcohol-precipitated QPs was an exothermic reaction.

### 2.9. Biological Activity of QPs

#### 2.9.1. Antioxidant Activities of QPs

The DPPH and ABTS radical scavenging capability have been widely used to investigate the total antioxidant activity of polysaccharides [31]. The scavenging activity of QPs on DPPH and ABTS radicals was presented in Table 3. As shown in Table 3, different components of quinoa polysaccharide all exhibited scavenging effects on ABTS and DPPH. QPE90 has the strongest ability to scavenge DPPH and ABTS radicals. The EC_50_ values were determined as 5.22 mg/mL, and 1.83 mg/mL, respectively. QPE50 has the weakest scavenging ability. The order of scavenging DPPH ability was QPE90 > QPE80 > QPE70 > QPE50 > QPE60. The order of scavenging ABTS ability was QPE90 > QPE70 > QPE80 > QPE60 > QPE50. From the above results, we could see that QPE90 showed the strongest antioxidant activity.

#### 2.9.2. Alpha-Amylase and Alpha-Glucosidase Inhibitory Activities of QPs

Alpha-amylase and alpha-glucosidase inhibitors could delay the release of glucose or fructose in the small intestine, and subsequently reduce postprandial hyperglycaemia [32]. Therefore, these inhibitors may be promising oral hypoglycemic agents for preventing type 2 diabetes. As shown in Table 3, different components of QPs all exhibited antidiabetic activity. QPE90 has the best inhibitory effects on α-amylase and α-glucosidase. The IC_50_ values were determined as 48.67 mg/mL and 61.36 mg/mL. QPE50 has the lowest inhibitory effects. The order of α-amylase inhibitory activity was QPE90 > QPE80 > QPE70 > QPE50 > QPE60, and the order of α-glucosidase inhibitory activity was QPE90 > QPE70 > QPE80 > QPE60 > QPE50. From the above results, we could see that QPE90 showed the best antidiabetic activity.

#### 2.9.3. Glycemic Index of QPs

GI is an indicator to measure the effect of carbohydrates on blood glucose levels, and is also a tool for type II diabetes diet management [33,34]. When GI is equal to, or higher than, 70 at the glucose level, carbohydrate foods are regarded as high GI foods that can be quickly digested and absorbed. While, when GI is equal to, or lower than, 55 at the glucose level, carbohydrate foods are considered low GI foods that digested and absorbed slowly. Low GI foods could reduce postprandial blood glucose levels, and this knowledge can be used to recommend and plan diets for people with diabetes [35].

As can be seen from Table 3, except for QPE50, all other QPs have a GI lower than 70. The GI value order of QPs was QPE50 > QPE60 > QPE80 > QPE70 > QPE90, which was significantly negatively correlated with the α-glucosidase inhibitory activity. α-glucosidase and α-amylase are key enzymes for carbohydrate digestion in the diet, and inhibitors of these enzymes may effectively delay the absorption of glucose [36]. Inhibition of α-glucosidase could slow down the decomposition of disaccharides into simple glucose, thereby reducing the amount of glucose absorbed in the blood, which affects the GI. This laid the foundation for the hypothesized mechanism of α-amylase and α-glucosidase inhibitors to reduce the GI. Therefore, we can conclude that the hypoglycemic potential of QPs is associated with their GI value and inhibition of α-amylase and α-glucosidase.

## 3. Materials and Methods

### 3.1. Materials

Quinoa seeds were purchased from Dulan, Qinghai province (China). Phenol, sulfuric acid, carbazole, bovine serum albumin, coomassie blue G-250, phosphoric acid, ethanol (China national pharmaceutical group, Beijing, China), chloroform, monosaccharide control (glucose, galactose, arabinose, xylose, mannose, rhamnose, ribose and fucose were purchased from Sino Pharm Chemical Reagent Co., Ltd. (Beijing, China), glucuronic acid and galacturonic acid were purchased from Sigma Co., Ltd., St. Louis, MO, USA). All other reagents were analytically pure.

### 3.2. Extraction and Isolation of Quinoa Polysaccharide (QPs)

**Figure 8:**
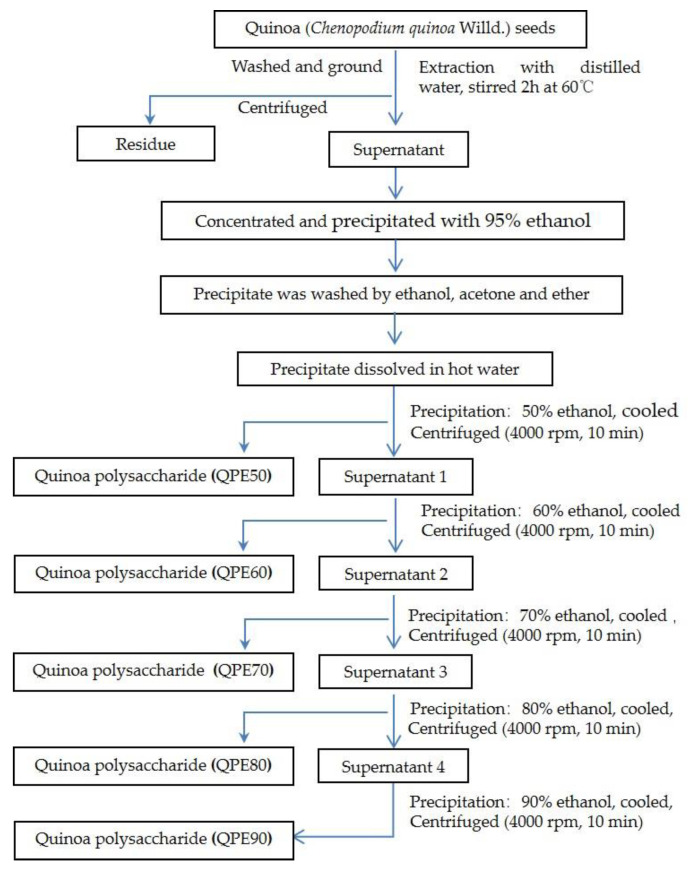
Flow chart of extraction and *isolation* of polysaccharides from quinoa.

One hundred gram quinoa seeds powder and 1000 mL distilled water were added in a container, the extraction was performed at 60 °C for 2 h, and repeated twice. The filtrates were combined, concentrated by a vacuum rotary evaporator, and precipitated with 3 vol of 95% ethanol at 4 °C for 12 h, followed by centrifugation at 4000 rpm for 10 min. The precipitate was washed by ethanol, acetone and ether, and then dissolved in hot water. Quinoa polysaccharides (QPs) were fractionated by using the gradient ethanol precipitation method. The scheme is illustrated in Figure 8. Briefly, ethanol was added into the solution to make an ethanol concentration of 50%, and followed by a refrigeration at 4 °C for 12 h, centrifugation and freeze-dry to obtain Quinoa polysaccharide (QPE50). For the supernatant of QPE50, ethanol was added into the solution to make an ethanol concentration of 60%, then refrigeration, centrifugation and freeze-dry to obtain QPE60. QPE70, QPE80, and QPE90 were prepared by gradient ethanol in sequence.

### 3.3. Structure and Physicochemical Properties of QPs

#### 3.3.1. Determination of Polysaccharide Yield, Chemical Composition and Average Molecular Weight of QPs

The content of total sugar in QPs was determined by the phenol-sulfuric acid method using glucose as the standard [37]. The protein content was determined by photometric assay according to the Bradford method using bovine serum albumin (BSA) as the standard [38]. The uronic acid content was determined by a carbazole-sulfuric acid method using galacturonic acid as the standard [39].

Polysaccharide yield is calculated by the following formula,

ω_1_(%) = 100 × m_1_/m_2_ (1)

where ω_1_ (%) is the polysaccharide yield, m_1_ (mg) is the weight of QPs, and m_2_ (mg) is the weight of quinoa seed used for extraction.

The high performance gel permeation chromatography (HPGPC-RID, Agilent1260, Santa Clara, CA, USA) equipped with a TSKgel G4000 pwXL column (7. 8 mm × 300 mm, 8 μm) was used to measure the molecular weight of QPs according to the method described by Zhu et al. (2019) [40]. The detector temperature was 35 °C, the mobile phase was deionized water, and the flow rate was 0.8 mL·min^−1^. Dextran standards with different molecular weights (T-10, T-25, T-40, T-70, T-500, and T-2000) were used to establish the standard curve for calibration.

#### 3.3.2. Monosaccharide Composition Analysis

The assay for monosaccharide composition was carried out according to the method of Dai with some modifications [41]. The polysaccharide sample (10 mg) was placed in a hydrolysis tube and hydrolyzed with 1.0 mL of 2.0 M trifluoroacetic acid (TFA) at 120 °C for 2 h, and then dried under nitrogen. Afterwards, 1 mL of 0.5 M 1-phenyl-3-methyl-5-pyrazolone (PMP) methanol solution and 0.5 mL of 0.3 M NaOH solution were added to the hydrolysate to be derived in a 70 °C water bath for 2 h. After cooling to room temperature, 0.5 mL of 0.3 M HCl solution was added, then 0.5 mL of chloroform was added for extraction, shaken thoroughly, and then allowed to stand for 20 min. The lower layer was discarded, and the upper layer was extracted twice with chloroform to obtain derivative products.

The derivative products were analyzed by HPLC (Agilent 1260 HPLC, Santa Clara, CA, USA) with a diode array detector and a C_18_ column (4.6 mm × 250 mm i.d., 5 μm, Waters). The eluted mobile phase was 0.1 M KH_2_PO_4_ solution (pH = 6.8, solvent A) and acetonitrile (solvent B), and used at 82% A and 18% B. The flow rate was 1.0 mL/min, the column temperature was 25 °C, and the detection wavelength was at 245 nm. Monosaccharides glucose, galactose, xylose, mannose, ribose, rhamnose, glucuronic acid, galacturonic acid, arabinose and fucose were used as references.

#### 3.3.3. Congo Red Test

The Congo red test of QPs was analyzed by spectrophotometry method [42]. The sample solution (0.5 mg/mL) was mixed with 2 mL, 50 µM Congo red and 1 M NaOH to achieve a final NaOH concentration of 0–0.50 M. At the same time, a mixed solution without polysaccharide was prepared as the control. The UV-vis absorption spectrum was measured in the range of 200 to 700 nm after equilibration at room temperature for 10 min.

#### 3.3.4. UV-VIS Spectroscopy and Fourier Transform Infrared Spectroscopy (FT-IR)

Each QPs sample was prepared with a mass concentration of about 2 mg·mL^−1^, and the spectroscopic analysis was performed at 200–600 nm by a UV-VIS spectrophotometer (Unico, Shanghai, China).

FT-IR spectra of QPs was analysed. All samples within KBr pellet were determined using FT-IR (TENSER 27, Bruker, Karlsruhe, Germany) with a spectral range of 4000 to 400 cm^−1^.

#### 3.3.5. Scanning Electron Microscopy

The surface microstructure of QPs was observed by a scanning electron microscopy (JSM-6700F, JEOL, Tokyo, Japan). The specimens were fixed on a metal stub with silver conductive tape, and were sputtered with gold using a sputter coater.

#### 3.3.6. Atomic Force Microscope (AFM) Observation

The purified QPs were dissolved in distilled water to a solution concentration of 10 μg/mL. 10 μL of the sample solution was dropped on the surface of cleaved mica (φ1 cm), and dried at 25 °C. Atomic force microscope (AFM, Bruker, Karlsruhe, Germany) was used for the determination of QPs morphology in the ambient condition under tapping mode [14].

#### 3.3.7. XRD Analysis

The X-ray diffraction patterns of the QPs were measured by a Rigaku Smartlab X-ray diffractometer equipped with a Cu-Kβ radiation source. The operating conditions of the diffractometer were: step size 0.01°, scan speed 15° min^−1^, tube pressure 40 kv, tube flow 200 mA, angular range of 5–90° (2θ).

#### 3.3.8. Thermal Analysis

Thermogravimetric analyzer (TG-DTA 6300, Tokyo, Japan) was used to investigate the weight loss stages of QPs. In the TGA experiment, the sample powders are heated from 50 to 800 °C with a heating rate of 10 °C/min under the nitrogen atmosphere. The weight of the sample was less than 5 mg to avoid the possible temperature gradient inside the sample and ensure the kinetic control of the process.

### 3.4. Determination of Biological Activity

#### 3.4.1. DPPH Radical Scavenging Activity

The DPPH free radical scavenging activity of QPs was measured according to previous method [43] with some modifications. Three milliliter of freshly prepared DPPH (0.1 mM in 50% ethanol) is added as a free radical source to 1 mL containing various concentrations (0, 0.25, 0.5, 0.75, 1.0, 2.0, 5.0, 10.0 and 20.0 mg/mL). The mixture was shaken rapidly for 5 min and incubated at 25 °C for 25 min. Vc was used as a positive control, and the absorbance was measured at 517 nm. The capability to scavenge the DPPH radical was calculated, using the following equation,

(2) $$\;\mathrm{DPPH}\;\mathrm{scavenged}\;\left(\%\right)=\left(1-\frac{\mathrm{As}\;-\;\mathrm{Asb}}{\;\mathrm{Ac}\;-\;\mathrm{Acb}}\;\right)\;\times \;100$$

where Ac represents the absorption of the negative control (DPPH with solvent), Acb represents the absorption of the system background (50% ethanol with solvent), As represents the absorption of the DPPH system with added sample, and Asb represents the absorption of the sample background (50% ethanol with sample). From a plot of concentration against % scavenging activity, a linear regression analysis was performed to determine the EC_50_ value of the sample.

#### 3.4.2. ABTS Radical Scavenging Activity

The 2,2′-azino-bis(3-ethylbenzothiazoline-6-sulfonic acid) (ABTS) radical cation (ABTS^+^•) scavenging activity of QPs was measured based on reported method [44]. Briefly, the ABTS solution (7 mM) was mixed with potassium persulfate solution (2.45 mM) in the dark for 12–16 h. The prepared ABTS solution was diluted 40–50 times with phosphate buffer (pH 7.4), to make its absorbance of 0.70 ± 0.02 at 734 nm. Then 0.4 mL of various concentrations (0, 0.25, 0.5, 0.75, 1.0, 2.0, 5.0, 10.0 and 20.0 mg/mL) of sample solution were added to 3 mL of ABTS^+^• solution. The solution was shaken rapidly until the absorbance became stable within 10 min at room temperature. The absorbance was measured at 734 nm, and Vc was used as a positive control. The capability to scavenge the ABTS radical was calculated based on the following equation,

(3) $$\;\mathrm{ABTS}\;\mathrm{scavenged}\;\left(\%\right)=\left(1-\frac{\mathrm{As}\;-\;\mathrm{Asb}}{\;\mathrm{Ac}\;-\;\mathrm{Acb}}\;\right)=\;\times \;100$$

where Ac represents the absorption of the negative control (ABTS with solvent), Acb represents the absorption of the system background (phosphate buffer with solvent), As represents the absorption of the ABTS system with added sample, and Asb represents the absorption of the sample background (phosphate buffer with sample). The EC_50_ value of QPs samples was obtained from a linear regression analysis of concentration against % scavenging activity.

#### 3.4.3. Alpha-Amylase Inhibitory Activity

The α-amylase inhibitory activity of QPs was determined according to the reported method [45] with slight modifications. Briefly, 40 μL of 0.2% (w/v) soluble starch, 20 μL of QPs solution or 0.1 M phosphate buffer (control) containing 6 mM CaCl_2_ (pH 6.9) and 20 μL of α-amylase dissolved in the above buffer were mixed in a 96-well plate to start the enzyme reaction. The reaction was stopped by adding 80 μL of 0.4 M HCl after incubating at 37 °C for 10 min. The absorbance was read at 580 nm after 100 μL of 5 mM I_2_ in 50 mM KI was added. Acarbose was used as positive control. The inhibitory activity of α-amylase was calculated as follows,

(4) $$\;\mathrm{inhibition}\;\left(\%\right)=\left(1-\frac{A_{4}\;-\;A_{3}}{\;A_{2}\;-\;A_{1}}\;\right)=\;\times \;100$$

where A_1_ and A_2_ were the absorbance of mixture containing starch and α-amylase or starch only. A_3_ and A_4_ were the absorbance of mixture containing starch and QPs with or without α-amylase. Inhibitory activity was expressed as IC_50_ value.

#### 3.4.4. Alpha-Glucosidase Inhibitory Activity

The inhibitory activity to α-glucosidase of QPs was evaluated according to the published method [46] with some modifications. A mixture of 150 μL of QPs solution and 100 μL of 0.1 M sodium phosphate buffer (pH = 6.7) containing α-glucosidase (0.1 U/mL) was incubated at 37 °C for 10 min. Then, 200 μL of 1 mM p-nitrophenyl-α-d-glucopyranoside (pNPG) solution dissolved in 0.1 M sodium phosphate buffer (pH = 6.7) was added into the mixture after pre-incubation. The reaction mixture was incubated 30 min at 37 °C. After incubation, the reaction was stopped by adding 1.0 mL 0.1 M Na_2_CO_3_, and the absorbance was measured at 405 nm. Acarbose was used as positive control. The inhibitory activity to α-glucosidase of QPs was calculated as follows,

(5) $$\;\mathrm{inhibition}\;\left(\%\right)=\left(1-\frac{A_{3}\;-\;A_{4}}{\;A_{1}\;-\;A_{2}}\;\right)=\;\times \;100$$

where A_1_ and A_2_ were the absorbance of control and control blank, A_3_ and A_4_ were the absorbance of sample and sample blank, respectively. Inhibitory activity was expressed as IC_50_ value.

#### 3.4.5. Glycemic Index of QPs

The glycemic index of QPs were determined according to the method of Li et al. [47] by the NutraScan GI 20 equipment (Next Instruments Pty Ltd., New South Wales, Australia). The GI value was calculated as the percentage of available carbohydrates converted to glucose when the incubation time reaches 240 min.

#### 3.4.6. Statistical Analysis

All the results were expressed as the mean ± standard deviation of triplicate measurements. Statistical analysis was performed using SPSS 19.0 for Windows (SPSS, Inc., Chicago, IL, USA). Statistical significances were carried out by one-way analysis of variance (ANOVA), followed by Duncan’s test. Values of p < 0.05 were considered as statistically significant.

## 4. Conclusions

In the present study, QPs was prepared by hot water extraction and further fractionated by gradient ethanol precipitation into five fractions of QPE50, QPE60, QPE70, QPE80 and QPE90. The polysaccharide contents were 52.82%, 63.69%, 67.15%, 44.56% and 41.01%, the protein contents were 11.13%, 8.54%, 9.50%, 7.01% and 3.32% and the Mw were 13,785 Da, 6489 Da, 4732 Da, 3318 Da and 1960 Da, respectively. HPLC and IR analysis showed that the five fractions of QPs were composed mainly of glucose. SEM and AFM results showed that QPs samples were mainly in spherical shape, and the intermolecular crosslinking degree and interaction force decreased with the increase in ethanol concentration Among the QPs, QPE90 showed a significantly higher antioxidant and antidiabetic activities than the other QPs. This study confirmed that the antioxidant and antidiabetic activity increase with the decrease of molecular weight of quinoa polysaccharides. It also confirmed that ethanol precipitation is an effective and rapid way to isolate polysaccharides with antioxidant and antidiabetic ability from quinoa polysaccharide. The GI of QPs was significantly correlated with the α-glucosidase inhibitory activity using correlation analysis of antidiabetic activity. This indicates that quinoa has the potential to develop into hypoglycemic products.

## Figures and Tables

**Table 1:** Yield, chemical composition and molecular weight of QPs precipitated by gradient ethanol.

Index	Samples				
	QPE50	QPE60	QPE70	QPE80	QPE90
Yield (w %)	0.84 ± 0.03	1.95 ± 0.02	2.26 ± 0.31	1.45 ± 0.26	0.74 ± 0.02
Total sugar (w %)	52.82 ± 0.02	63.69 ± 0.07	67.15 ± 1.32	44.56 ± 1.32	41.01 ± 2.58
Uronic acid (w %)	20.84 ±1.23	21.51 ± 1.21	31.30 ± 1.62	19.61 ± 1.22	25.88 ± 1.16
Protein (w %)	11.13 ± 0.69	8.54 ± 1.06	9.50 ± 0.75	7.01 ± 0.74	3.32 ± 0.08
Average Molecular Weights (Da)					
Mw	13,785	6489	4732	3318	1960
Mn	8265	5206	4082	2943	1689
Mw/Mn	1.67	1.25	1.16	1.13	1.16

**Table 2:** Monosaccharide composition of different QPs components.

Monosaccharide Composition	Molar Composition (mol%)				
	QPE50	QPE60	QPE70	QPE80	QPE90
l-Rhamnose	-	0.73 ± 0.04	-	-	-
d-Glucose	94.37 ± 1.25	87.92 ± 2.56	92.21 ± 2.57	100 ± 1.27	100 ± 1.79
d-Galactose	-	4.67 ± 0.39	2.98 ± 0.03	-	-
d-Arabinose	5.63 ± 0.06	6.68 ± 0.05	4.81 ± 0.04	-	-

**Table 3:** Antioxidant, antidiabetic activities and glycemic index of QPs.

Quinoa Polyssacharides	Antioxidant activity		Antidiabetic Activity		Glycemic Index (GI)
	DPPH EC_50_(mg/mL)	ABTS EC_50_(mg/mL)	α-Amylase IC_50_ (mg/mL)	α-Glucosidase IC_50_ (mg/mL)	
QPE50	13.67 ± 0.59b	5.21 ± 0.27a	102.66 ± 0.98b	92.38 ±0.66a	72.19 ± 0.51a
QPE60	15.22 ± 0.50a	2.39 ± 0.05b	105.73 ± 1.60a	81.79 ± 0.77b	68.37 ± 0.39b
QPE70	8.19 ± 0.57c	2.13 ± 0.06b	82.17 ± 0.95c	53.26 ± 1.02d	65.40 ± 0.35c
QPE80	8.06 ± 0.53c	2.22 ± 0.12b	72.99 ± 0.90d	57.28 ± 0.59c	65.92 ± 0.37c
QPE90	5.22 ± 0.47d	1.83 ± 0.03b	61.36 ± 0.58e	48.67 ± 0.65e	61.68 ± 0.22d
Vc	0.01 ± 0.00e	0.01 ± 0.00c	-	-	-
Acarbose	-	-	2.65 ± 0.32f	0.23 ± 0.01f	-

## Abbreviations

QPs	Quinoa polysaccharides
QPE50	Quinoa polysaccharide precipitated with 50% ethanol
QPE60	Quinoa polysaccharide precipitated with 60% ethanol after QPE50
QPE70	Quinoa polysaccharide precipitated with 70% ethanol after QPE60
QPE80	Quinoa polysaccharide precipitated with 80% ethanol after QPE70
QPE90	Quinoa polysaccharide precipitated with 90% ethanol after QPE80
FT-IR	Fourier Transform Infrared Spectroscopy
SEM	Scanning electron microscope
AFM	Atomic force microscope
XRD	X-ray diffractometry
TG-DTA-DTG	Thermogravimetry-Differential Thermal Analysis-Derivative thermogravimetry
